# Chlorhexidine Prevents Root Dentine Mineral Loss and Fracture Caused by Calcium Hydroxide over Time

**DOI:** 10.1155/2017/1579652

**Published:** 2017-04-30

**Authors:** Michael Ranniery Garcia Ribeiro, Érika Bárbara Abreu Fonseca Thomaz, Darlon Martins Lima, Tarcísio Jorge Leitão, José Bauer, Soraia De Fátima Carvalho Souza

**Affiliations:** ^1^Post-Graduate Program of Dentistry, Federal University of Maranhão (UFMA), São Luis, MA, Brazil; ^2^Department of Public Health, Federal University of Maranhão (UFMA), São Luis, MA, Brazil; ^3^Department of Dental Materials, Federal University of Maranhão (UFMA), São Luis, MA, Brazil; ^4^Department of Pediatric Dentistry, Federal University of Maranhão (UFMA), São Luis, MA, Brazil; ^5^Department of Endodontics, Federal University of Maranhão (UFMA), São Luis, MA, Brazil

## Abstract

*Purpose*. To evaluate the mineral ion loss of root dentine after treatment with 2% chlorhexidine solution (CHX) and to compare its yield and flexural strength (fs) after exposure to calcium hydroxide [Ca(OH)_2_].* Materials and Methods*. Dentine bars (DB) were made from 90 roots of bovine incisors and randomized into three groups: G_Control_: distilled/deionized water (DDW), G_NaOCl_: 2.5% sodium hypochlorite + 17% EDTA, and G_CHX_: CHX + DDW. The release of phosphate (PO_4_) and calcium (Ca) ions was measured by spectrophotometry. The DB were exposed to Ca(OH)_2_ paste for 0, 30, 90, and 180 days. DB were subjected to the three-point bending test to obtain yield and fs values. The fracture patterns were evaluated (20x). Data were analyzed using Kruskal-Wallis and Dunn's post hoc tests or one- and two-way ANOVA followed by Tukey's post hoc test (*α* = 0.05).* Results*. G_CHX_ showed lower PO_4_^3−^ and Ca^2+^ ionic release than G_NaOCl_ (*p* < 0.001). For yield and fs, G_CHX_ > G_NaOCl_ in all periods (*p* < 0.001), except for yield strength values on 90 days (*p* = 0.791). A larger frequency of vertical fractures was observed in G_NaOCl_ and that of oblique fractures in G_CHX_ (*p* < 0.05).* Conclusions.* CHX prevented PO_4_^3−^ and Ca^2+^ loss and showed a tendency to preserve the yield and fs of root dentine over time following exposure to Ca(OH)_2_ paste.

## 1. Introduction

 In endodontic practice, the need to treat young permanent teeth with immature root walls beyond open root apex is routine [1, 2]. Therefore, amidst new treatment techniques, such as pulp regeneration [3, 4], calcium hydroxide remains as intracanal medication of choice in these cases [5].

Calcium hydroxide [Ca(OH)_2_] is an osteoinductive substance that is able to induce the formation of an apical biological barrier on nonvital immature permanent teeth [1]. Moreover, some studies warn that its prolonged use may negatively affect the mechanical properties of dentine by its dissolution effect on organic matrix, exposing the teeth to clinical fractures [6, 7].

During endodontic treatment, sodium hypochlorite solution (NaOCl) has been used to assist in the cleaning and shaping of root canal systems. Ethylenediaminetetraacetic acid (EDTA) has been employed due to its chelating action on mineral ions and smear layer removal [8, 9]. Hydroxyapatite, the main inorganic constituent of root dentin, is mainly composed of phosphorus and calcium. Scientific findings have shown that EDTA may change the superficial composition of human root dentine via decreases in phosphorous [4] and calcium content [8], and NaOCl may degrade the dentinal organic matrix, modifying its mechanical properties [10–12].

To maintain the mechanical properties of teeth with physiologically immature roots, chlorhexidine digluconate solution (CHX) has been recommended as an endodontic irrigating agent, as it promotes the integrity of the collagen fibrillar network, inhibiting the action of dentinal organic matrix metalloproteinases (MMPs) [13, 14]. Moreover, CHX has immediate antimicrobial properties, high substantivity, and relatively low toxicity [11, 15]. The alterations of cumulative effect related to the use of endodontic irrigation agents and intracanal medication may negatively influence the mechanical properties of endodontically treated teeth, which may incur radicular fractures [16].

Thus, it was hypothesized that the treatment of root dentine with 2% CHX solution could be an alternative to NaOCl, avoiding the removal of mineral content and minimizing the deleterious effects caused by prolonged exposure to Ca(OH)_2_ paste on the mechanical properties of root dentine.

Therefore, the aims of this study were to quantify the concentration of inorganic phosphate (PO_4_) and calcium (Ca) ions released from root dentine after treatment with 2.5% NaOCl or 2% CHX and to compare the yield and flexural strength of root dentine treated with 2.5% NaOCl or 2% CHX exposed to Ca(OH)_2_ for 30, 90, and 180 days.

## 2. Materials and Methods

### 2.1. Specimen Preparation

Ninety bovine incisor teeth with total length of 25 mm (±5 mm) were decoronated in the cement-enamel junction by a metallographic saw (Isomet 1000, Buehler Ltd, Lake Bluff, IL, USA) for the preparation of dentine tubes from the roots (10 mm ± 2 mm), which were cut into two dentine plates of 1 mm thickness each. The plates were longitudinally sectioned in each millimeter (±0.2 mm), forming 4 to 8 dentine bars (DB) of 1 × 1 × 10 mm, measured with a digital paquimeter (Mitutoyo, Tokyo, Japan). The DB of each root were stored in polypropylene tubes with 1.5 mL of distilled and deionized water (DDW) for 24 h.

The roots were randomly distributed into three groups: G_Control_: DDW, G_NaOCl_: 2.5% NaOCl + 17% EDTA, and G_CHX_: 2% CHX + DDW. The DB of G_NaOCl_ and G_CHX_ were distributed into four subgroups according to the Ca(OH)_2_ exposure period (no exposure and 30, 90, and 180 days). The polypropylene tubes with DB were filled by 0.675 g of Ca(OH)_2_ paste (Calen; SS White, Rio de Janeiro, RJ, Brazil; composition: 2.5 g of calcium hydroxide; 0.5 g of zinc oxide; 0.05 g of hydrogenated colophony; 1.65 mL of polyethyleneglycol 400) and kept at 37°C and 100% relative humidity. The DB were solution-treated (Farmácia Garrido, São Luis, MA, Brazil) according to their groups, in an ultrasonic cleaning device (BioFree Gnatus, Ribeirão Preto, SP, Brazil), under standardized volume (1.5 mL). Vibration time and solution change conditions are shown in Table 1. The pH of the solutions were measured in triplicate (Digimed, Campo Grande, MS, Brazil), being DDW = 6.4, 2.5% NaOCl = 11.1, 17% EDTA = 4.4, and 2% CHX = 7.4.

### 2.2. PO_4_ and Ca Concentration

In order to determine PO_4_ and Ca concentration in irrigating agents (NaOCl, CHX, and DDW) after each treatment, an aliquot of 100 *μ*L was collected for PO_4_ and 10 *μ*L for Ca dosage after each wash. Then, a colorimetric analysis was conducted, with Arsenazo III and phosphomolybdate as reagents (Doles, Goiânia, GO, Brazil) by multiplate spectrophotometer (Elx800UV, Biotek Instruments, Winooski, VT, USA) at a wavelength of 630 nm, in triplicate [17]. The patterns were prepared with the same composition as the samples. The EDTA solution was not evaluated due to its chelating effect on colorimetric agents.

### 2.3. Three-Point Bending Test

To obtain yield and flexural strength data, the three-point bending fixture test was employed (Odeme, Joaçaba, SC, Brazil) while DB were mounted on a universal testing device (Instron 3342, Canton, MA, USA). Loading was applied to DB under a crosshead speed of 0.5 mm/min. The established leaking limit for the dentinal substrate was 2% of permanent deformation. The yield strength data (MPa) were directly obtained from the tension × deformation graph (Instron Bluehill, Canton, MA, USA). The flexural strength values were calculated using the following equation: *σF* = 3*FL*/2*b* · *h*^2^, where *σ* is the flexural strength (MPa), *F* is the necessary load for fracture, *L* is the distance between the supports (6 mm), and *b* and *h* are the test specimen's width and height (mm), respectively.

### 2.4. Cohesive Fracture Pattern Analysis

In order to determine the nature of fractures, the fragments were aligned and photographed with a digital camera (Q-Color 5, Olympus America Inc., Center Valley, PA, USA), adapted to a stereomicroscope (SZ61, Olympus Latin America Inc., Miami, FL, USA) at 20x magnification. The fracture patterns were divided into three types: vertical fracture, when the fracture line occurred parallel to the force application axis; oblique fracture, when the fracture line occurred transversally to the force application axis; and no rupture, when the fracture occurred with no fragment separation.

### 2.5. Statistical Analysis

For the statistical analysis, the DB from the same root were considered as experimental units. The Shapiro-Wilk test was applied for the evaluation of data distribution. The comparative analysis of variables among groups and times was processed using Kruskal-Wallis and Dunn's post hoc tests for nonparametric data or one- and two-way ANOVA with Tukey's post hoc test on parametric data.

Differences in the absolute and relative frequencies (%) of the fracture patterns due to irrigating agent types and to the period were evaluated by *χ*^2^ and *χ*^2^ trend. The DB in which fragment separation did not occur were excluded from the analysis: G_Control_ = 4; G_NaOCl_ = 19; and G_CHX_ = 36. All analyses were performed with Stata software (version 11.0, StataCorp, TX, USA) and BioEstat (version 5.3, Mamirauá Institute, Tefé, AM, Brazil). The established significance level was 5%.

## 3. Results

### 3.1. PO_4_ and Ca Concentration

**Figure 1:**
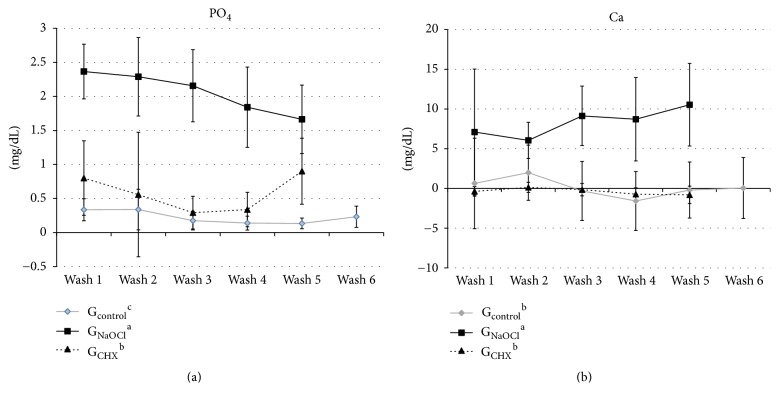
Ion concentration (mg/dL) of phosphate (PO_4_) and calcium (Ca) released from the DB by the irrigating agents. Different lowercase letters indicate statistical difference among groups.

As the irrigating agents were being replaced, a reduction in PO_4_ ions was observed (*p* = 0.016), which did not occur with Ca (*p* = 0.939; Figure 1). The concentration of PO_4_ was higher in G_NaOCl_ > G_CHX_ > G_Control_ (*p* < 0.001; Figure 1(a)) and the concentration of Ca was higher in G_NaOCl_ than in the other groups (*p* < 0.001). No significant difference was observed in Ca concentration between G_CHX_ and G_Control_ (*p* = 0.536; Figure 1(b)).

### 3.2. Yield and Flexural Strength

**Figure 2:**
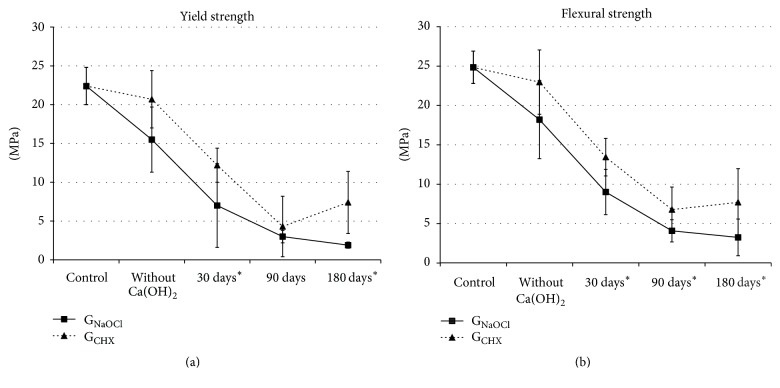
Yield and flexural strength. G_NaOCl_: 2.5% NaOCl + 17% EDTA + Ca(OH)_2_. G_CHX_: 2% CHX + DDW + Ca(OH)_2_. ^*∗*^Statistical difference among exposure time (*p* < 0.001).

A tendency toward a reduction in yield and flexural strength values was observed, regardless of the irrigating agent applied (*p* < 0.001; Figure 2). G_CHX_ showed higher yield and flexural strength values than G_NaOCl_ in all evaluated periods (*p* < 0.001; Figure 2(a)), except for the yield strength values at 90 days (*p* = 0.791).

### 3.3. Cohesive Fracture Pattern Analysis

**Figure 3:**
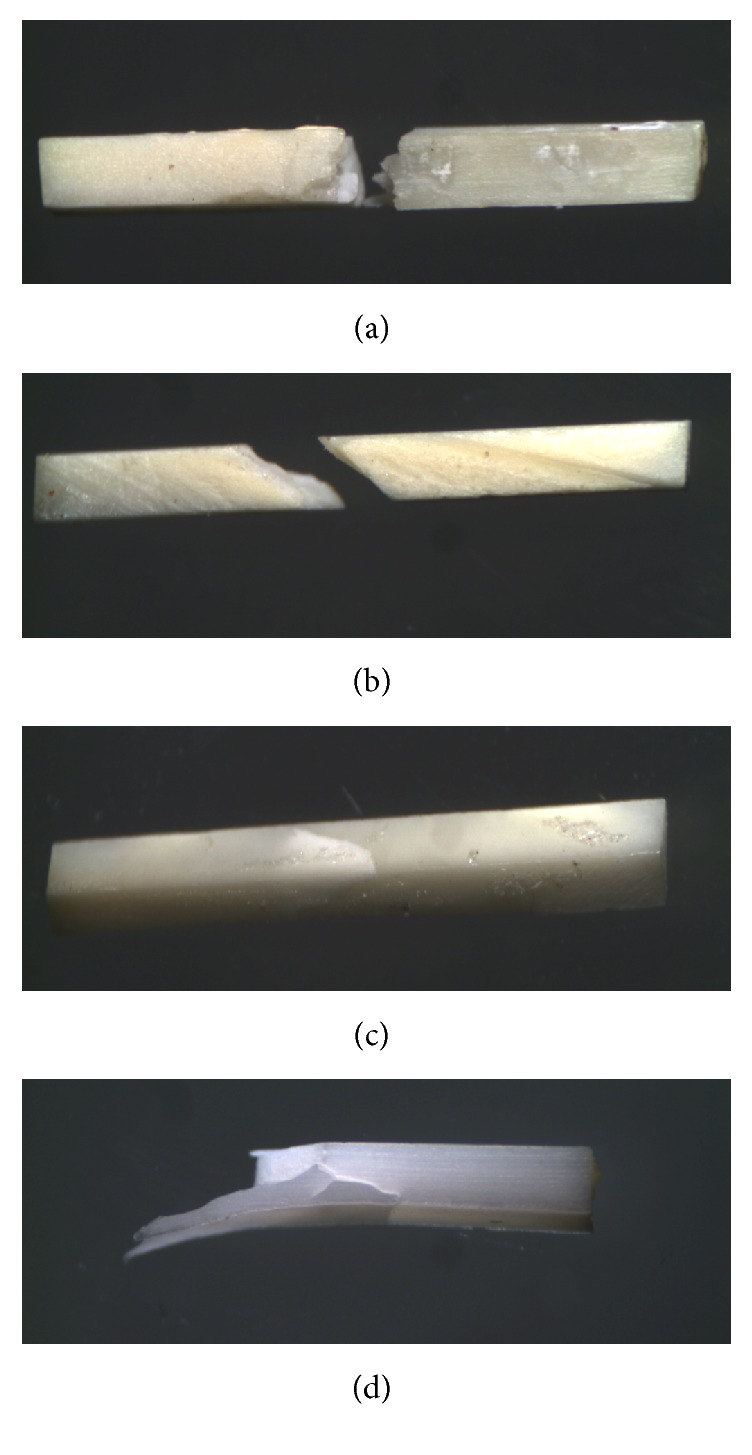
Pattern of cohesive fractures (20x): (a) vertical fracture; (b) oblique fracture; (c) no rupture; (d) greenstick-type oblique fracture.

A difference was observed in the fracture pattern among the experimental groups of the 180-day period only (*p* = 0.031). A tendency toward an increase in vertical fractures was observed in G_NaOCl_ (*p* = 0.018; Table 2). Eighteen oblique, greenstick-like fractures were identified in G_CHX_ (Figure 3).

## 4. Discussion

Root dentine treatment with chlorhexidine prevents PO_4_ and Ca ion loss, and after exposure to calcium hydroxide paste for up to 180 days, it shows a protective synergistic effect on the cohesive resistance of dentine, which was verified through the large yield and flexural strength values, compared under the same conditions, when irrigated with sodium hypochlorite [18] followed by EDTA. The irrigation protocol based on the use of NaOCl and EDTA contrasts with the protocol that uses chlorhexidine and water [19], mainly by the disintegration of organic tissue and substantivity of chlorhexidine. However, depending on the conditions of temperature, concentration, and time of use, this disintegration goes beyond the remaining pulp tissue and weakens the root dentin.

Some studies have shown that chlorhexidine acts on the maintenance of root dentine integrity, owing to PO_4_ and Ca ionic fixation [20, 21] and to the proteolytic inhibition capacity of MMPs 2, 8, and 9 [22], which reduces demineralized organic matrix degradation [20]. Accordingly, Ferrer-Luque et al. [23] verified the absence of dentine decalcification by chlorhexidine solution at 2% using atomic absorption spectrophotometry. Sodium hypochlorite at ≥2.5%, thanks to its powerful mineral ion uptake capacity, removes the organic content from dentine and denatures the collagen fibers by fragmentation of long peptide chains and chlorinated protein terminal groups [24]. Besides the effect of sodium hypochlorite solution, demineralizing effect of EDTA also arises, leading to a decrease in the mechanical properties of dentine [6]. Such a process seems to have occurred in this experiment, as G_NaOCl_ removed the majority of PO_4_ and Ca ions from DB when compared with G_CHX_ or G_Control_ (Figure 1; *p* < 0.001). Ca removal remained while the denaturation of dentin treated with NaOCl during washes, characteristic result of sequestration of this ion after radicular dentin denaturation [3, 6, 9].

The DB exposed to calcium hydroxide presented higher flexural strength values for G_CHX_ than for G_NaOCl_ in all experimental periods (Figure 2(b)). This phenomenon showed the tendency of chlorhexidine to maintain this property over time (*p* < 0.05), preventing the morphological structural change of the dentine organic matrix, which is responsible for most of the mechanical properties of this substrate [25, 26]. Moreover, this finding corroborates the lower mineral loss identified in this group.

The yield strength analysis indicates a permanent and irreversible substrate deformation range (offset at 2%). Such a dentine substrate alteration may be clinically translated as early radicular fractures of the endodontically treated tooth, especially on immature teeth, which present thin and fragile dentine walls [27]. By understanding that the yield strength indicates an initial range of permanent deformation, it may be speculated that, from this point on, the formation of microcracks may occur and may lead to catastrophic root fractures during physiological mastication movements. The smaller the distance between the yield and flexural strength values, the more friable the dentine substrate. In this sense, the results of this study point to approximate values for these properties in both experimental groups over time, perhaps due to a loss of dentine elasticity, which can be attributed to the alkalizing action of calcium hydroxide [4, 7, 28].

As stated in the study of Sim et al. [18], the DB subjected to the three-point-bending test in this experiment behaved as friable materials. This was verified in some specimens, in which yield strength values coincided with the maximum flexural strength values, showing absence of plastic deformation. This phenomenon is attributed to the fact that a critical alkaline challenge was created on all DB surfaces for a long period. However, Marending et al. [9] maintained that, for the mechanical properties analysis of the dentine substrate, the use of DB allows more accurate results. In this study, even when exposed to this alkaline challenge, the chlorhexidine-treated DB presented a tendency of the collagen fibrillar network to remain united during the mechanical test. It is believed that the maintenance integrity of the collagen fibrillar network that characterizes the chlorhexidine-treated dentine elasticity [15, 29] functioned as a protective factor against fractures.

The DB wash time was established considering the average time spent during endodontic treatment and intracanal medication changes in cases of apexification. The use of EDTA after the 2.5% NaOCl solution as well as distilled water solution after 2% CHX was assigned to traditional treatment with these irrigating solutions [12, 15, 18].

The result of fracture pattern analysis in G_CHX_ was predominantly oblique (Figure 3(b)), sometimes with no fragmentation (Figure 3(c); *n* = 36 DB). To ratify this collagen elasticity, another variation in the oblique fracture pattern, likely to the greenstick pattern, was observed (Figure 3(d); *n* = 18 DB), similar to the findings of Grigoratos et al. [6].

Still on the fracture pattern analysis, the vertical fracture tendency increase over time for NaOCl-treated DB points to an increase in rigidity and consequent dentine substrate weakness due to the long-term exposure to calcium hydroxide (Table 2; Figure 3(a)). The collagen component is coresponsible for the resistance and hardness of hard tissues [30] and once exposed to Ca(OH)_2_, this collagen matrix may be modified into a more mineralized dentine, resulting in a more fragile and less resistant, yet harder substrate [28].

As used by Kawamoto et al., the bovine model used in the present study is a biological model already consolidated in the literature, as it provides a better standardization of the dentin age, it is easy to obtain teeth in good condition, and it shows less variability in organic/inorganic composition than human teeth [31].

It is worth mentioning that the DB were obtained in the longitudinal direction of the cervical and middle region of the roots. However, it was not sought to standardize the positioning of the DB on flexural strength device. The average values obtained by root for each parameter were considered, considering nonstandardization position of the DB dentinal tubules. This care was taken because there is no calculable effect of the dentinal tubules orientation on the elastic behavior of the natural dentin [32, 33]. Thus, it is believed that the arrangement of the dentinal tubules did not interfere in the results of this study. Hence, it is believed that the selection of the irrigating agent must be pondered for the success of long-term endodontic treatment of immature teeth, considering that the increase in concentration, volume, and exposure period to NaOCl may develop permanent deleterious effects on this still-forming radicular dentine. Furthermore, previous studies point to the effective antibacterial activity of chlorhexidine over the endodontic microbiota [34, 35] and its role in the structural protection of dentine [29, 36].

Future clinical studies could be verified, in this line of thought, if the endodontic treatment of teeth with physiologically immature roots using chlorhexidine at 2% as an irrigation agent may help in the prevention of catastrophic radicular fractures.

## 5. Conclusion

It may be concluded that the root dentine substrate treatment with chlorhexidine prevented mineral loss, and when dentine was exposed to calcium hydroxide paste for a long time, it tends to preserve its yield and flexural strengths.

## Figures and Tables

**Table 1:** Type of irrigation agent, vibration time (between washes), and exposure to Ca(OH)_2_.

Group	Irrigating agents	Exposure to Ca(OH)_2_
	Vibration time [solution's replacement time]	Time (days)
G_Control_ (*n* = 10)	DDW 30 min [5 min]	—
G_NaOCl_ (*n* = 40)	2.5% NaOCl + 17% EDTA 150 min [30 min] + 5 min [1 min]	Without Ca(OH)_2_ 30, 90, and 180
G_CHX_ (*n* = 40)	2% CHX + DDW 150 min [30 min] + 30 min [5 min]	Without Ca(OH)_2_ 30, 90, and 180

—: negative control.

**Table 2:** Cohesive fracture pattern absolute and relative frequency distribution (%).

	Groups							
	G_NaOCl_ + Ca(OH)_2_				G_CHX_ + Ca(OH)_2_			
	Time (days)				Time (days)			
Fracture pattern	Without Ca(OH)_2_	30	90	180^‡^	Without Ca(OH)_2_	30	90	180^‡^
Vertical	19^d^	24^c^	26^b^	44^a^	16^a^	22^a^	18^a^	17^a^
	36.5%	50%	57.8%	72.1%	36.4%	48.9%	45.0%	50.0%
Oblique	33^a^	24^b^	19^c^	17^d^	28^a^	23^a^	22^a^	17^a^
	63.5%	50%	42.2%	27.9%	63.6%	51.1%	55.0%	50.0%

Total	52	48	45	61	44	45	40	34
*p* values	0.018^*∗*^				0.952^*∗*^			

^*∗*^ *χ* ^2^ trend. ^**‡**^*χ*^2^ test (*p* = 0.031). ‡ indicates statistical difference among the same periods of time between groups. Different lowercase letters indicate statistical difference for the same treatment group and fracture pattern.

## References

[1] Chala S., Abouqal R., Rida S. (2011). Apexification of immature teeth with calcium hydroxide or mineral trioxide aggregate: systematic review and meta-analysis. *Oral Surgery, Oral Medicine, Oral Pathology, Oral Radiology and Endodontology*.

[2] Skaare A. B., Jacobsen I. (2005). Primary tooth injuries in Norwegian children (1–8 years). *Dental Traumatology*.

[3] Yang J., Yuan G., Chen Z. (2016). Pulp regeneration: current approaches and future challenges. *Frontiers in Physiology*.

[4] Yassen G. H., Eckert J. E., Platt J. A. (2015). Effect of intracanal medicaments used in endodontic regeneration procedures on microhardness and chemical structure of dentin. *Restorative Dentistry & Endodontics*.

[5] Abbott P. V. (1998). Apexification with calcium hydroxide—when should the dressing be changed? The case for regular dressing changes. *Australian Endodontic Journal*.

[6] Grigoratos D., Knowles J. C., Ng Y. L., Gulabivala K. (2001). Effect of sodium hypochlorite and calcium hydroxide on the modulus of elasticity and flexural strength of dentin. *International Endodontic Journal*.

[7] Yassen G. H., Platt J. A. (2013). The effect of nonsetting calcium hydroxide on root fracture and mechanical properties of radicular dentine: a systematic review. *International Endodontic Journal*.

[8] Doğan H., Çalt S. (2001). Effects of chelating agents and sodium hypochlorite on mineral content of root dentin. *Journal of Endodontics*.

[9] Marending M., Paqué F., Fischer J., Zehnder M. (2007). Impact of irrigant sequence on mechanical properties of human root dentin. *Journal of Endodontics*.

[10] Cehreli Z. C., Uyanik M. O., Nagas E., Tuncel B., Er N., Comert F. D. (2013). A comparison of residual smear layer and erosion following different endodontic irrigation protocols tested under clinical and laboratory conditions. *Acta Odontológica Scandinávica*.

[11] Pascon F. M., Kantovitz K. R., Sacramento P. A., Nobre-dos-Santos M., Puppin-Rontani R. M. (2009). Effect of sodium hypochlorite on dentine mechanical properties. A review. *Journal of Dentistry*.

[12] Ximenes M., Triches T. C., Beltrame A. P. C. A., Hilgert L. A., Cardoso M. (2013). Effect of endodontic irrigation with 1% sodium hypochlorite and 17% EDTA on primary teeth: a scanning electron microscope analysis. *General Dentistry*.

[13] Buzalaf M. A. R., Kato M. T., Hannas A. R. (2012). The role of matrix metalloproteinases in dental erosion. *Advances in Dental Research*.

[14] Kim D.-S., Kim J., Choi K.-K., Kim S.-Y. (2011). The influence of chlorhexidine on the remineralization of demineralized dentine. *Journal of Dentistry*.

[15] Gomes B. P. F. A., Vianna M. E., Zaia A. A., Almeida J. F. A., Souza-Filho F. J., Ferraz C. C. R. (2013). Chlorhexidine in endodontics. *Brazilian Dental Journal*.

[16] Zelic K., Vukicevic A., Jovicic G., Aleksandrovic S., Filipovic N., Djuric M. (2015). Mechanical weakening of devitalized teeth: three-dimensional finite element analysis and prediction of tooth fracture. *International Endodontic Journal*.

[17] Vogel G. L., Chow L. C., Brown W. E. (1983). A microanalytical procedure for the determination of calcium, phosphate and fluoride in enamel biopsy samples. *Caries Research*.

[18] Sim T. P. C., Knowles J. C., Ng Y.-L., Shelton J., Gulabivala K. (2001). Effect of sodium hypochlorite on mechanical properties of dentine and tooth surface strain. *International Endodontic Journal*.

[19] Mohammadi Z., Abbott P. V. (2009). The properties and applications of chlorhexidine in endodontics. *International Endodontic Journal*.

[20] Kato M. T., Leite A. L., Hannas A. R. (2012). Impact of protease inhibitors on dentin matrix degradation by collagenase. *Journal of Dental Research*.

[21] Scaffa P. M. C., Vidal C. M. P., Barros N. (2012). Chlorhexidine inhibits the activity of dental cysteine cathepsins. *Journal of Dental Research*.

[22] Martin-De Las Heras S., Valenzuela A., Overall C. M. (2000). The matrix metalloproteinase gelatinase A in human dentine. *Archives of Oral Biology*.

[23] Ferrer-Luque C. M., Perez-Heredia M., Baca P., Arias-Moliz M. T., Gozález-Rodríguez M. P. (2013). Decalcifying effects of antimicrobial irrigating solutions on root canal dentin. *Medicina Oral, Patología Oral y Cirugía Bucal*.

[24] Davies J. M. S., Horwitz D. A., Davies K. J. A. (1993). Potential roles of hypochlorous acid and N-chloroamines in collagen breakdown by phagocytic cells in synovitis. *Free Radical Biology and Medicine*.

[25] Carrilho M. R. O., Carvalho R. M., De Goes M. F. (2007). Chlorhexidine preserves dentin bond in vitro. *Journal of Dental Research*.

[26] Moreira D. M., Affonso Almeida J. F., Ferraz C. C. R., de Almeida Gomes B. P. F., Line S. R. P., Zaia A. A. (2009). Structural analysis of bovine root dentin after use of different endodontics auxiliary chemical substances. *Journal of Endodontics*.

[27] Yassen G. H., Chin J., Mohammedsharif A. G., Alsoufy S. S., Othman S. S., Eckert G. (2012). The effect of frequency of calcium hydroxide dressing change and various pre- and inter-operative factors on the endodontic treatment of traumatized immature permanent incisors. *Dental Traumatology*.

[28] Yassen G. H., Vail M. M., Chu T. G., Platt J. A. (2013). The effect of medicaments used in endodontic regeneration on root fracture and microhardness of radicular dentine. *International Endodontic Journal*.

[29] Hebling J., Pashley D. H., Tjäderhane L., Tay F. R. (2005). Chlorhexidine arrests subclinical degradation of dentin hybrid layers in vivo. *Journal of Dental Research*.

[30] Wang X., Bank R. A., TeKoppele J. M., Mauli Agrawal C. (2001). The role of collagen in determining bone mechanical properties. *Journal of Orthopaedic Research*.

[31] Kawamoto R., Kurokawa H., Takubo C., Shimamura Y., Yoshida T., Miyazaki M. (2008). Change in elastic modulus of bovine dentine with exposure to a calcium hydroxide paste. *Journal of Dentistry*.

[32] Kinney J. H., Habelitz S., Marshall S. J., Marshall G. W. (2003). The importance of intrafibrillar mineralization of collagen on the mechanical properties of dentin. *Journal of Dental Research*.

[33] Mannocci F., Pilecki P., Bertelli E., Watson T. F. (2004). Density of dentinal tubules affects the tensile strength of root dentin. *Dental Materials*.

[34] Onçağ Ö., Hoşgör M., Hilmioğlu S., Zekioğlu O., Eronat C., Burhanoğlu D. (2003). Comparison of antibacterial and toxic effects of various root canal irrigants. *International Endodontic Journal*.

[35] Siqueira J. F., Rôças I. N., Paiva S. S. M., Guimarães-Pinto T., Magalhães K. M., Lima K. C. (2007). Bacteriologic investigation of the effects of sodium hypochlorite and chlorhexidine during the endodontic treatment of teeth with apical periodontitis. *Oral Surgery, Oral Medicine, Oral Pathology, Oral Radiology and Endodontology*.

[36] Rahimi S., Janani M., Lotfi M. (2014). A review of antibacterial agents in endodontic treatment. *Iranian Endodontic Journal*.

